# Scientometric Analysis of The Relationship between a Built Environment and Cardiovascular Disease

**DOI:** 10.3390/ijerph19095625

**Published:** 2022-05-05

**Authors:** Zhonghui Zheng, Ping Zhang, Fangzheng Yuan, Yunque Bo

**Affiliations:** 1 School of Architecture & Art Design, Hebei University of Technology, Tianjin 300132, China; 201922301004@stu.hebut.edu.cn (Z.Z.); a17302220763@163.com (F.Y.); 2 Policy Research Department, Tianjin Medical Information Center, Tianjin 300041, China; puyixibo@163.com

**Keywords:** built environment, cardiovascular disease, scientometric analysis, walkability, physical activity, food environment

## Abstract

The prevention and treatment of cardiovascular disease (CVD) are necessary to improve patient quality of life and to reduce the burden of medical and other social problems. Reducing the impact of CVD through environmental intervention was hailed as the most economical approach and research into such interventions is becoming key. The purpose of this article is to summarize the research topics and developments in the field of the built environment and CVD between 2000 and 2021 using scientometric analysis. In total, 1304 records retrieved from the Web of Science core database were analyzed using CiteSpace software, and the results were displayed using knowledge mapping. The number of publications and conferences relating to the built environment and CVD showed an upward trend over the study period, with the United States taking the lead. Physical activity and the food environment were used as mediators and entry points to map the relationship between the built environment and CVD. Walkability, residence characteristics, the food environment, and greenness were key research topics. Research shifted over the period to incorporate quantitative analyses of subjective feelings while focusing on decreasing sedentary behavior. Understanding the variability in the built environment is critical to improving the generalizability of the findings presented in the individual studies. Inter-disciplinary and multi-disciplinary research is conducive to innovation and ensuring the integration of real environmental elements. This study provides an overview and valuable guidance for researchers relating to how the built environment impacts CVD.

## 1. Introduction

Over the past three decades, the prevalence of all cardiovascular diseases (CVD) has nearly doubled from 271 million in 1990 to 523 million in 2019, while the number of deaths from CVD has shown a steady increase from 12.1 million in 1990 to 18.6 million in 2019, accounting for approximately one-third of global deaths each year [1,2]. The global trend for disability-adjusted life-years (DALYs) and years of life lost (YLLs) attributed to CVD has also increased significantly, with the number of years lived with disability (YLDs) doubling from 17.7 million to 34.4 million over the period [1,3].

There is an urgent need to prevent and treat CVD to improve patient quality of life and reduce the medical burden. The World Health Organization (WHO) proposed a global action plan in 2013, with the goal of reducing premature mortality from non-communicable diseases by 25% by the year 2025 [4]. It is hoped that in their efforts to achieve this goal, countries will focus their efforts on reducing the impact of CVD and its related risk factors.

Interventions to reduce the impact of CVD are primarily either at the individual or population levels [5,6]. Individual interventions typically involve the treatment of CVD using medications and are associated with a significant socioeconomic burden. Population interventions are comprehensive measures to reduce CVD morbidity and mortality by modifying the behaviors that predict risk factors for CVD [7,8]. Population interventions have a limited impact on the final indicator in the short term. However, such interventions are also associated with positive effects as observed through secondary outcomes such as diet and physical activity [9]. Population-level intervention is recognized by the WHO as the most economical intervention, being more cost-effective than individual interventions, and identified the built environment as the key area for population intervention [4].

Guiding healthy lifestyle choices through built environment intervention is recognized and actively supported by the policies and guidelines of relevant institutions globally [10,11,12,13,14,15,16,17,18]. The International Federation of Science and Technology has provided clear evidence for a significant relationship between CVD and the built environment, and proposed in 2003 that countries take active measures to improve public health by optimizing the built environment [19]. The American Heart Association (AHA) proposed the concept of “ideal cardiovascular health” in 2010, aiming to reduce the incidence and mortality of CVD in the USA. By 20% between 2010 and 2021 through environmental interventions to stimulate lifestyle changes [20]. The Active Communities Tool (ACT) from the USA Department of Health and Human Services, the Active Design Guidelines from Sport, England, and Planning by Design from Canada’s Planning Department have all emphasized the impact of physical space in the built environment for promoting healthy activity levels. Residents become healthier through environmental design that promotes walking and physical activity. A growing number of scholars recognize the potential for environmental interventions to have sustained and wide-ranging impacts, as well as the potential to promote significant changes in population-level physical activity [21].

Therefore, the purpose of this article is to use the CiteSpace tool to analyze the literature in the field of a built environment and CVD between 2000 and 2021. This article focuses on three aspects: (1) An examination of the current state of research in the field; (2) an examination of the relationship between the role of the built environment and CVD; and (3) an exploration of research hotspots and future trends in the field.

## 2. Materials and Methods

### 2.1. Data Source and Research Method

**Figure 1:**
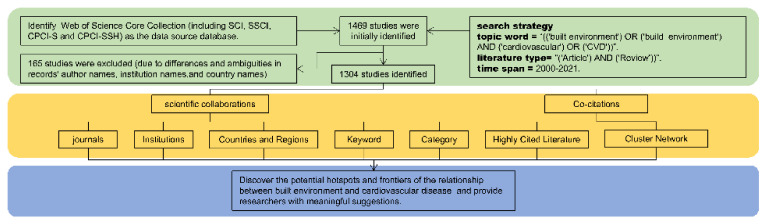
The flowchart of the study design.

This study used Web of Science, limiting the search scope to the metrics of Science Citation Index (SCI), Social Science Citation Index (SSCI), Conference Proceedings Citation Index-Science (CPCI-S), and Conference Proceedings Citation Index—Social Sciences and Humanities (CPCI-SSH). An advanced search was conducted using the subject terms (“(‘built environment’) OR (‘build environment’)” AND “(‘cardiovascular’) OR (‘CVD’)”) and literature type (“(‘Article’) And (‘Review’) AND (‘Proceedings Paper ’)”) across the date range 2000–2021. After preliminary searching, 1469 records were obtained. Due to differences and ambiguities in records’ author names, institution names, and country names, CiteSpace’s data deduplication and name merge functions were used to standardize the data. A total of 1304 unique records were finally obtained and included in the analysis. Figure 1 illustrates the literature screening process.

### 2.2. Data Visualization and Analysis

CiteSpace, developed by Drexel University’s Professor Chen Chaomei of Computer and Information Science, was used to visualize the structure, regularity, and distribution of the knowledge domain describing the relationship between built environment and CVD, as well as to analyze the co-citation of articles and mine the knowledge clustering and distribution of the citation space [22]. CiteSpace (5.6.R2) (Philadelphia, PA, USA) was used in this study to conduct a scientometric analysis and to visualize the results. The frequency with which two documents are cited together is defined as co-citation. To identify relationships such as collaborations between institutions and countries, a co-occurrence analysis was performed between knowledge units [23]. Finally, a relationship network and a research knowledge map were constructed based on the results of the scientometric analysis.

The scientometric analysis included parameters and methods for identifying knowledge maps. The knowledge map shows the age of the record using cool and warm colors, the colors are close to red as the date approaches 2021 [24]. The size of the nodes in the knowledge map represent the frequency of institutions, countries, and journals, and the connections between the nodes represent the existence of these nodes in the same article. There are also some parameter indicators for a specific evaluation in the process of scientometric analysis. H-index is used to evaluate the amount of academic output and the level of the scholarly output of researchers and institutions. H-index indicates that h of the N papers published in the journal were cited at least h times [22]. The degree indicates the number of connections between authors (institutions, countries) in the co-occurrence knowledge graph. A higher degree value indicates more communication and cooperation between the authors (institutions, countries). Centrality is an indicator that measures the importance of nodes in the research cooperation network [25]. In addition, “burst” value indicates that the literature has received significant attention for a certain time [22].

## 3. Results

### 3.1. Research Outputs

**Figure 2:**
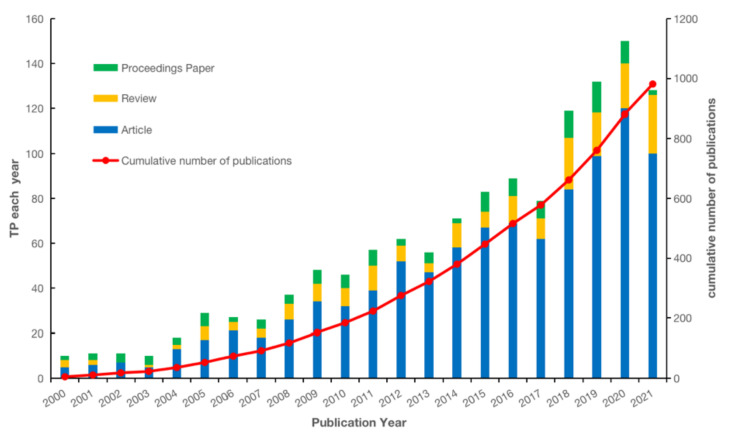
The scientific outputs from 2000–2021.

The variation in the number of research outputs reflects the changes in the interest of international experts and scholars in the field in describing the impact of the built environment on CVD. A total of 1304 research records were retrieved, including 956 articles, 202 reviews, and 146 conference papers. Figure 2 shows the scientific output. The number of published papers each year rose from 2004 to 2021. Overall, the global research output in the field of the built environment and CVD continues to rise. Specifically, three types of literature (articles, reviews, and conference papers) also showed increasing trends.

### 3.2. Scientific Collaborations

#### 3.2.1. Analysis of Journals

**Figure 3:**
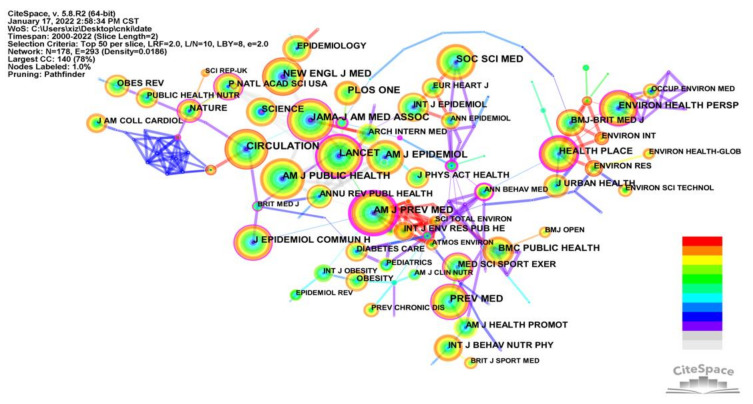
Knowledge map of co-citation journals network.

The cited frequency of the journal shows the frequency of research citations in the field from the journal and indicates the journal’s influence and importance in the field of studying the relationship between the built environment and CVD [26,27]. Figure 3 and Table 1 show a co-citation analysis of the journals. The top five core journals according to citation frequency, impact factor, centrality, and H-index were *CIRCULATION*, *AM J PREV MED*, *LANCET, AM J PUBLIC HEALTH* and *JAMA-J AM MED ASSOC.* The node circles of all these journals are all relative in Figure 3 and their importance in the field. Among them, both *AM J PREV MED* and *INT J ENV RES PUB HE* have a red trend in the connecting line and node color, which indicates their interest in the built environment and CVD field in recent years and their close collaboration with each other. Notably, six of the top ten journals in the field originated from the USA (*CIRCULATION*, *AM J PUBLIC HEALTH*, *JAMA-J AM MED ASSOC*, *PLOS ONE*, *NEW ENGL J MED*, and *PREV MED*) with three from the UK (*LANCET*, *AM J EPIDEMIOL*, and *SOC SCI MED*), and one from the Netherlands (*AM J PREV MED*).

#### 3.2.2. Collaborations between Institutions

**Figure 4:**
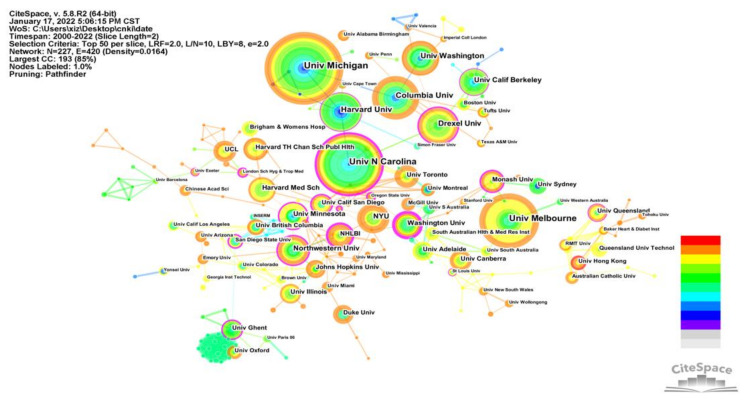
Knowledge map of co-institution collaboration network.

Figure 4 illustrates a network of collaborations comprising 227 nodes and 520 links reflecting collaborative exchange between institutions globally. The nodes from the USA are independent and have a larger radius, indicating that they are relatively independent of each other, but represent a significant volume of publications. In contrast, European institutions are smaller but more collaborative.

The top 20 institutions in terms of publications are listed in Table 2. The top five are the University of Michigan, the University of Washington, the University of North Carolina, the University of Melbourne, and the University of Columbia. It is worth noting that 15 of the top 20 institutions are from the USA, representing 14 schools and one government research institute. This is followed by three universities in Australia, and one each in the UK and Canada, further demonstrating the outstanding contribution and leadership of the USA in the field. The temperature of node colors on the map signify that the University of North Carolina, the NHLBI (US National Heart Lung and Blood Institute), and the University of Washington feature more in recent years.

#### 3.2.3. Collaborations between Countries and Regions

**Figure 5:**
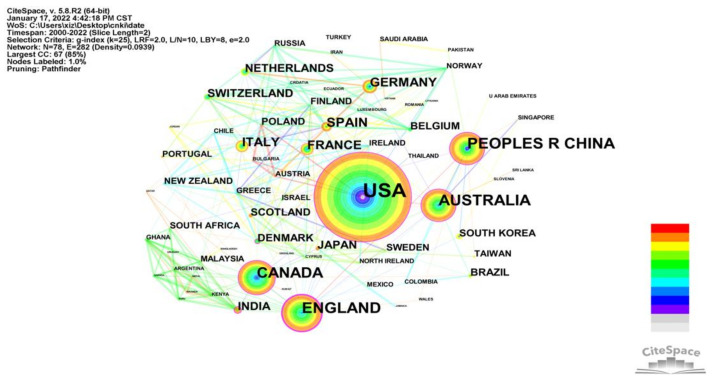
Knowledge map of the inter-country/inter-region network.

The network density of the inter-country/inter-region network map is 0.0939, with 78 nodes and 282 connecting lines. The higher the network density of the knowledge map, the more connections between countries and the closer the cooperation and exchange. This indicates that the relationship between the built environment and CVD research has attracted the attention of countries around the world. As shown in Table 3, the USA has the highest number of publications in the health field globally with 424 publications, accounting for 30.57% of the total. It is much higher than either China (95) or the UK (95), which are equal in second place. The other two countries in the top five are Australia (91) and Canada (87). Figure 5 shows the USA node to have both warm and cold colors, signifying its consistent performance over time in this area. Although China has published 95 articles and is in second place, none of the top 20 research institutions are from China. This indicates that although China is a late starter in the field, it is growing rapidly, and a large number of recent contributions to the field were made by scholars and institutions in China. The majority of outputs are from Western high-income countries whose built environment interventions are expected to achieve the WHO target of reducing premature deaths from non-communicable diseases by 25% by 2025. Conversely, low- and middle-income countries which already account for more than three-quarters of CVD deaths worldwide have less research in this area. The focus of CVD reduction in low- and middle-income countries is more on individual interventions, but the impact of CVD in these countries is dire due to limitations of resources and availability of healthcare, inadequate healthcare funding, and poor governance [28,29]. 

#### 3.2.4. Analysis of Co-Occurrence Keywords

**Figure 6:**
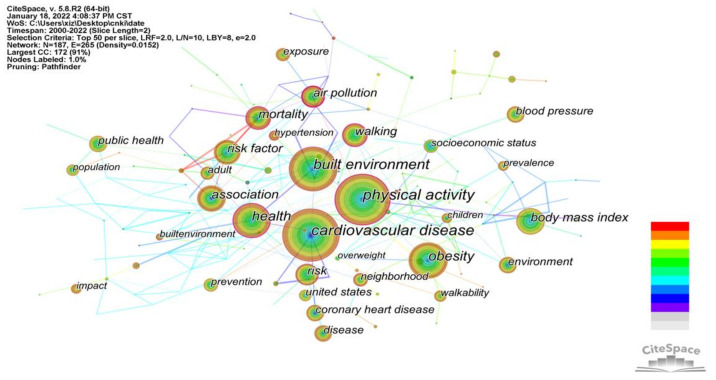
Knowledge map of analysis of co-occurring keywords.

Keyword analysis using keyword co-occurrences facilitates the identification of research hotspots in the field. Figure 6 shows the keyword co-occurrence analysis map where node size represents the frequency of keyword occurrences and connections between the nodes represent the co-occurrence of keywords in the same document. The more co-occurrences, the thicker the connections, and therefore the stronger the correlation between the keywords. As shown in Table 4, the top ten keywords in terms of frequency were physical activity, cardiovascular disease, built environment, obesity, health, association, mortality, body mass index, risk factor, and walking. The keywords used to measure the centrality of the elements analyzed were physical activity, cardiovascular disease, built environment, health, mortality, walking, risk, air pollution, and neighborhood. Relevant studies have paid the most attention to the main goal and direction of the built environment and CVD research, the mechanism by which the built environment impacts CVD, the prevention and treatment of CVD in specific groups, and built environment risk factors associated with CVD.

#### 3.2.5. Analysis of Co-Occurrence Category

**Figure 7:**
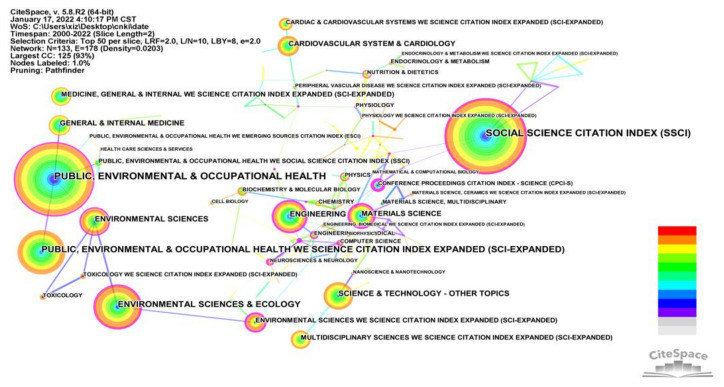
Knowledge map of co-occurrence categories.

The discipline classification in this study was taken from the Web of Science database. In Figure 7, a purple circle around the edge of a node circle represents a high intermediate centrality value for that node. The top ten disciplinary categories in terms of co-occurrence frequency are listed in Table 5. The main discipline categories involved in the study of the built environment and CVD are “Social Science Citation Index (SSCI)”, “Public, Environmental & Occupational Health”, “Public, Environmental & Occupational Health Web of Science Citation Index Expanded (Sci-Expanded)”, “Environmental Sciences & Ecology”, and “Environmental Sciences”. This analysis highlights that research in the built environment and CVD is highly multi-disciplinary in nature. 

### 3.3. Literature Co-Citations Analysis

#### 3.3.1. Highly Cited Literature

**Figure 8:**
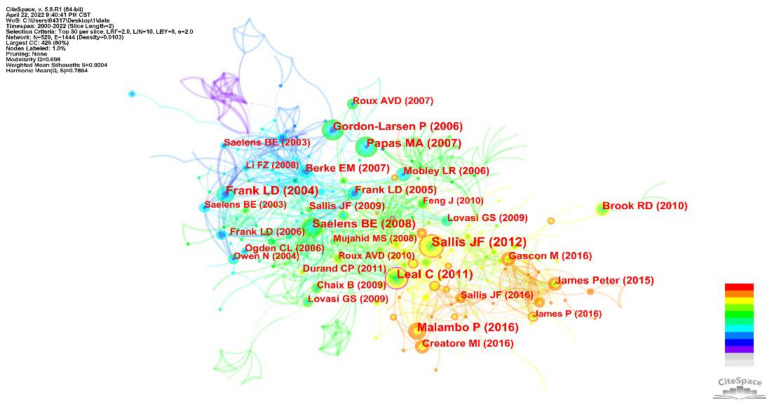
Knowledge map of co-citation literature.

The cross-referencing of scientific literature provides an objective reflection of scientific development in a field. The early citation network in Figure 8 is relatively dense and has a large node radius. The number of citations is high and concentrated in the period before 2010. The 10 most frequently cited papers were selected and shown in Table 6. It should be noted that the citation frequencies in this paper are limited to cross-citations between the 1304 articles included in the analysis, so the citation frequencies shown here differ from those provided by Web of Science. The article “*Role of Built Environments in Physical Activity, Obesity, and Cardiovascular Disease*” by JF Sallis was the most frequently cited.

The mechanisms and relationships between the built environment and CVD have not yet been agreed upon by the academic community. The multi-level ecological models of behavior developed by JF Sallis and applied to physical activity describe the key concepts and summarize the evidence surrounding the relationship between the built environment and CVD. The article provides recommendations for the development of an environment that promotes physical activity and improves CVD. The largest burst is in the “*Obesity Relationships with Community Design*, *Physical Activity, and Time Spent in Cars*” by Lawrence D. Frank of the University of California, USA. It was widely noted and discussed for its focus on the relationship between equity and accessibility in the built environment and obesity-induced CVD. Further, two of the top ten articles are written by this author, who is a leader in the study of public health and physical activity, with an H-index of 90.

#### 3.3.2. Cluster Analysis of the Literature Co-Citation Network

**Figure 9:**
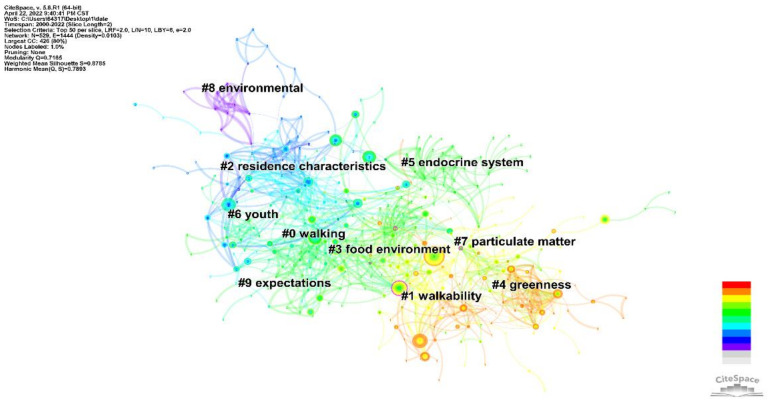
Knowledge map of literature cluster.

The knowledge map of the literature clustering is shown in Figure 9. The two measures of clustering effectiveness, Q = 0.7165 and S = 0.8785, indicate that the clusters formed have significant structure and are reasonably clustered. The color of the clustering blocks from cool to warm indicates the average time to cluster from past to present. The lower the cluster number, the more content it contains. To further understand the theme of clustering, we summarized the details of the clusters and plotted them in Table 7. All the clustering results have a profile value greater than 0.7, indicating that there are no problems with the clustering. 

Figure 9 and Table 7 show that walkability, active commuting, neighborhood, residence characteristics, and socioeconomics are the key themes in the field. 

Walking (cluster 0) and walkability (cluster 1) are the two clusters that have received the most attention, focusing on the impact of walkability in the built environment and on residents’ walking behavior and thus on cardiovascular health [39,40,41]. Walkability is an important component of physical activity and physical activity serves as a mediator between the built environment and CVD research. Numerous studies have used this concept as a breakthrough point. Walkability in the built environment is negatively associated with the prevalence of CVD and its risk factors [42,43,44,45]. Improving walkability can reduce the duration of sedentary time and increase physical activity levels among residents. This clustering study also addresses pedestrian infrastructure, safety, aesthetics, and noise. To reduce the risk of CVD, city planners can increase the amount of pedestrian infrastructure and the size of pavement buffers and improve the safety of streets [46,47,48]. Walkability as a cluster emerged later than walking, which reflects the development of research on walking behavior. Walking studies have focused on the measurement of pedestrian aggregates and thus quantitative descriptions of walking behavior, such as pedestrian flow in street sections and pedestrian indices. However, such indicators only consider the accessibility of facilities. More recently, scholars have integrated quantitative measures of spatial quality, using walkability as an important indicator to describe how the built environment can be used to promote walking [38,49,50,51,52,53,54,55].

Residence characteristics (cluster 2) are an important factor influencing CVD, which involves the satisfaction of residents’ basic needs along multiple dimensions and has an important impact on mental health [56]. Residence characteristics include biological, chemical, and physical dimensions, such as safety (crime rate, traffic accident rate, emergency shelter), health (air pollution, waste disposal rate, noise, drinking water standards), convenience (number and level of educational, medical, commercial, recreational facilities and children’s playgrounds), accessibility (number and quality of transport facilities and routes, distance from the city center), and comfort (number and size of parks, public open spaces, greening rate, building density, building heights, historical and cultural aspects of the neighborhood, residential identity) [57,58,59,60]. In addition to objective residential characteristics, several subjective variables including pride and satisfaction with their residential environment and indoor air quality were shown to be associated with CVD [61,62].

The food environment (cluster 3) influences cardiovascular health through its impact on residents’ travel activities and dietary energy intake. Studies have constructed corresponding evaluation indicators from the accessibility, availability, and affordability of food in the environment and analyzed their correlation with CVD risk factors [63]. An environment with good access to healthy foods reduces CVD risk factors. Each standard deviation increase in the hypertension rate is associated with a 12% reduction [64]. The level of access to healthy foods is positively associated with cardiovascular health [34,42,65]. Conversely, in residential environments where fast food intake is high, individual dietary control is relatively reduced [66]. Residents more readily access unhealthy food and consequently are at a higher risk of CVD [67,68,69,70]. The food environment has an indirect positive effect on physical activity and eating behavior, mainly through socio-economic and cultural factors that influence the cognitive abilities and material conditions of the population.

Greenness (cluster 4) describes the public green space available in the built environment. Green space effectively promotes physical activity and reduces air pollution. This reduces the morbidity and mortality of CVD and can be used as a place of recovery for CVD patients [71,72,73,74]. Studies have constructed quantitative indicators of green space (e.g., green space rate, quantity and quality of green space, accessibility of green space) to quantify the correlation with the prevalence of CVD. To do this, studies have reported the influence of various elements of green space on cardiovascular health, such as a negative correlation between green space rate, quantity, and accessibility of green space and the prevalence of CVD with a radius of 1–3 km [18,75,76,77,78,79,80]. In addition, the youth is a group that was identified as needing special attention. The China Cardiovascular Disease Report 2020 predicted that CVD among obese youths in China will increase rapidly in the next 10 years and reported that it has become a major negative influence on public health in China [81,82]. The decline in the frequency and duration of physical activity among the youth has led to an increase in health problems such as CVD and mental illness. To prevent CVD, it is recommended that youths should engage in at least 1 h of moderate to vigorous physical activity per day, and that lifestyle intervention should be intensified to reduce the amount of sedentary time spent and increase the amount of time spent undertaking physical activity.

## 4. Discussion

### 4.1. General Information

This study has shown that the literature in the research field describing the impact of the built environment on CVD shows an exponential growth trend, and its literature growth cycle is largely consistent with the global CVD crisis. The increase in the number of conference papers reflects, to some extent, a continued international interest of experts and scholars in the field and a recognition of the increasingly important role of the built environment in promoting physical activity and improving cardiovascular health in the population [10,12].

Much of the institutional and national research collaboration, as well as major co-cited journals in this field, has been concentrated in the USA and Europe. The Universities of Michigan and Washington in the USA are outstanding contributors to the field. The most co-cited journals are *CIRCULATION* (in the USA), *AM J PREV MED* (in The Netherlands), and *LANCET* (in the UK), all of which are highly regarded internationally and have played significant roles in the history of human public health. The USA and Europe are leading the way in this field and, as a result of their early research and interventions in CVD, the mortality rate from CVD in Europe and the USA has been declining in recent years [83]. However, of the 2 million deaths from CVD worldwide each year, more than half occur in low- to middle-income countries and regions [1]. Because of their limited access to healthcare and inadequate health funding, there is an urgent need to strengthen research in low- to middle-income countries in the future to promote global cardiovascular health at low cost through the most cost-effective built environment interventions for CVD. 

### 4.2. Research Topics

This study focuses on the hot research topics and emerging trends in the field of the built environment and CVD research in terms of keyword co-occurrence analysis and literature co-citation analysis. The findings described five main research topics in the field, namely the main goal and direction of the built environment and CVD research, the main mechanism of impact of the built environment on CVD, the prevention and treatment of CVD in specific groups of people, built environment risk factors that affect CVD, and measures of how the built environment affects CVD.

The main objectives in the study of the relationship between the built environment and CVD were health promotion, chronic disease prevention, and palliative care. Several systematic and narrative reviews have identified physical activity and the food environment as mediating variables and research entry points between the built environment and CVD.

Empirical studies have used behavioral pathways as mediating variables for the effect of the built environment on CVD, and reduced built environment risk factors that affect CVD, including type 2 diabetes, obesity, overweightness, body mass index, and cardiometabolic risk, by undertaking behavioral interventions, including increasing physical activity and improving the availability of healthy food. The main elements within the built environment found to affect CVD were the six areas of the walking environment, the food environment, the sports and entertainment environment, the active commuting environment, the public space environment, and the living environment. The field also focused on the prevention and treatment of CVD in specific groups of people and the impact of macroeconomic policies on the built environment.

### 4.3. Emerging Trends

The initial development phase of the field (before 2007) focused on key elements of the built environment in the form of individual human subjects, such as walking. In the transitional phase of development (2007–2013), the keywords built environment were more likely to reflect human perceptions and became suitable for quantitative analysis. For example, ‘walkability’ is a more scientific representation of the capacity for walking around an environment than ‘walking’ and is more suitable for quantitative research using spatial data. In the multi-dimensional development phase (2014 to present), with the advancement of society and technology, the way people live and work has changed significantly. Research into cardiovascular health has shifted from having an isolated focus on physical activity to having a concurrent focus on the harmful association between sedentary time and heart health, emphasizing both that sedentary time is poor for health and highlighting the difficulties of increasing the duration of daily physical activity.

The following issues need to be considered to move research in this field forwards:

Design prospective studies for the built environment and CVD. Scientific research is dedicated to promoting a shift from sedentary behavior in work, study, life, or recreation to more active behavior, which also requires improvements in the built environment to be achieved progressively. At the same time, future research should emphasize the physiological suitability of users in terms of the quality of spatial structure, for example, the “green view rate” is a more scientific representation of the experience of the pedestrian environment than the “greening rate” or “green space rate”.

Global variability in the built environment is regarded as an important factor. To compensate for the limitations of previous studies, which heavily emphasized Western countries with large land areas, low population densities, and sparse populations, future research should focus on the relationship between extremes of built environment attributes and CVD in high-density areas. Increasing variability in built environment settings, even within a single study, is critical for understanding dose–response relationships and improving the generalizability of research findings.

Further interdisciplinary research initiatives involving public environmental science, building engineering science, social science, medicine, toxicology, and computing researchers are necessary to disentangle the complex relationships between the built environment and CVD. Elements of the built environment co-exist and interact in the real environment. This co-existence and interaction of all possible built environment elements can be taken into account to more accurately predict the relative contribution of each individual built environment element to the CVD, and a simulated built environment assessment model using computers and big data needs to be established. In particular, the ability to draw on research methods and mechanisms from other disciplines has facilitated the advancement of quantitative research on the built environment and CVD. It has also been possible to keep pace with scientific developments using big data and artificial intelligence.

### 4.4. Strengths and Limitations

The current study has several advantages. This study dissects the hotspots and frontiers in the field of built environment and CVD research in a systematic manner. Our findings offer promising recommendations for future research on CVD-friendly built environments and highlight existing issues in the literature. To address the global CVD crisis, our study provides research directions for promoting healthy lifestyles through built environment construction. 

Despite the study’s positive findings, there are some limitations. In terms of the literature data, the literature data in this article are sourced solely from the Web of Science core collection database. Furthermore, we only chose documents written in English. Second, there is no grey literature in this article, such as non-publicly published government documents, dissertations, non-publicly issued conference documents, scientific reports, technical archives, and so on. This article does not interpret all of the information in the knowledge graph from the standpoint of visual analysis. It is also one of the problems and directions that the follow-up research should consider and investigate further.

## 5. Conclusions

This study conducted a scientometric analysis of the knowledge structure and knowledge domains of research in the field relating to the built environment and its impact on CVD based on data from 1304 records retrieved from the Web of Science core collection from 2000 to 2021. A visual analysis of the knowledge units in the field was conducted to create a comprehensive knowledge map. There was found to be extensive research in the field, involving multi-disciplinary theories and methods, and its development has involved the participation of researchers and scholars from various fields. Low- and middle-income countries, where CVD currently has a high prevalence, still need to strengthen their research in this area. This study provides a systematic and comprehensive analysis of the scientific results, key institutions and countries, high-impact journals, research collaboration networks, research themes, and emerging trends in the field. This article provides researchers with a global health overview in the form of an up-to-date, comprehensive, and holistic knowledge map. This study contributes to the existing built environment and CVD research and provides valuable guidance for researchers in the field.

## Figures and Tables

**Table 1:** The top 20 journals.

Rank	Journal	Cited Frequency	Impact Factor	Centrality	H-Index
1	CIRCULATION.	352	14.065	0.07	570
2	AM J PREV MED.	330	3.651	0.45	193
3	LANCET.	329	44.862	0.24	700
4	AM J PUBLIC HEALTH.	323	5.380	0.00	236
5	JAMA-J AM MED ASSOC.	289	51.270	0.18	622
6	AM J EPIDEMIOL.	266	4.287	0.05	234
7	SOC SCI MED.	265	0.030	0.00	213
8	PLOS ONE.	253	2.942	0.00	268
9	NEW ENGL J MED.	251	40.148	0.07	933
10	PREV MED.	250	4.011	0.12	154
11	HEALTH PLACE.	239	3.900	0.31	89
12	J EPIDEMIOL COMMUN H.	236	3.892	0.11	152
13	BMC PUBLIC HEALTH.	230	2.560	0.02	117
14	ENVIRON HEALTH PERSP.	221	8.326	0.12	249
15	SCIENCE.	177	41.030	0.00	1058
16	INT J BEHAV NUTR PHY.	172	7.460	0.04	95
17	INT J EPIDEMIOL.	167	7.276	0.02	183
18	MED SCI SPORT EXER.	162	4.053	0.12	203
19	P NATL ACAD SCI USA.	156	9.580	0.20	699
20	INT J ENV RES PUB HE.	156	3.180	0.00	78

**Table 2:** The top 20 institutions.

Rank	Institution	Cited Frequency	Impact Factor	Centrality
1	Univ. Michigan	38	0.05	5
2	Washington Univ.	32	0.64	10
3	Univ. N Carolina	32	0.47	5
4	Univ. Melbourne	29	0.02	3
5	Columbia Univ.	23	0.07	4
6	Harvard Univ.	21	0.14	6
7	Drexel Univ.	19	0.31	5
8	Northwestern Univ.	16	0.21	9
9	NYU	16	0	1
10	Univ. Calif Berkeley	15	0.11	7
11	Univ. Minnesota	14	0.24	7
12	Harvard Med. Sch.	13	0.06	4
13	Harvard TH Chan Sch. Publ. Hlth.	12	0.02	2
14	Univ. Toronto	12	0.03	5
15	NHLBI	12	0.47	6
16	Univ. Canberra	11	0	2
17	Monash Univ.	11	0.27	8
18	Johns Hopkins Univ.	11	0.09	8
19	Univ. Illinois	11	0.07	7
20	UCL	11	0.07	7

**Table 3:** The top 20 countries.

Rank	Country	Publications	Percent %	Centrality	Degree
1	USA	424	30.57%	0.12	14
2	PEOPLES R CHINA	95	9.87%	0.13	12
3	ENGLAND	95	9.87%	0.24	22
4	AUSTRALIA	91	9.45%	0.2	18
5	CANADA	87	9.03%	0.15	16
6	SPAIN	44	4.57%	0.09	19
7	FRANCE	40	4.15%	0.04	13
8	ITALY	39	4.05%	0	9
9	GERMANY	38	3.95%	0.05	12
10	INDIA	28	2.91%	0.11	15
11	JAPAN	25	2.60%	0.04	7
12	NETHERLANDS	25	2.60%	0.06	13
13	SWITZERLAND	24	2.49%	0.05	15
14	DENMARK	21	2.18%	0.16	21
15	BELGIUM	18	1.87%	0.01	11
16	SCOTLAND	18	1.87%	0.03	10
17	BRAZIL	17	1.77%	0	4
18	SOUTH KOREA	16	1.66%	0.01	5
19	POLAND	16	1.66%	0.03	13
20	SWEDEN	14	1.45%	0.06	11

**Table 4:** The top 20 keywords.

Rank	Keyword	Frequency	Centrality	Degree	Burst
1	physical activity	234	0.37	7	-
2	cardiovascular disease	233	0.16	8	-
3	built environment	192	0.13	3	-
4	obesity	136	0.08	3	-
5	health	134	0.26	8	-
6	association	99	0.06	4	-
7	mortality	90	0.21	9	-
8	body mass index	86	0.06	4	-
9	risk factor	77	0.04	4	-
10	walking	77	0.23	7	-
11	risk	70	0.12	5	-
12	air pollution	69	0.35	7	-
13	coronary heart disease	50	0	1	4.92
14	blood pressure	50	0	1	-
15	disease	49	0.04	3	-
16	united states	49	0	2	-
17	public health	49	0.04	4	-
18	environment	47	0.01	3	-
19	exposure	46	0	1	-
20	neighborhood	42	0.12	5	-

**Table 5:** The top 10 subject categories.

Rank	Category	Frequency	Centrality	Burst
1	SOCIAL SCIENCE CITATION INDEX (SSCI)	357	0.21	-
2	PUBLIC, ENVIRONMENTAL & OCCUPATIONAL HEALTH	315	0.27	-
3	PUBLIC, ENVIRONMENTAL & OCCUPATIONAL HEALTH WE SCIENCE CITATION INDEX EXPANDED (SCI-EXPANDED)	175	0	-
4	ENVIRONMENTAL SCIENCES & ECOLOGY	148	0.36	-
5	ENVIRONMENTAL SCIENCES	90	0.34	-
6	SCIENCE & TECHNOLOGY—OTHER TOPICS	78	0.06	-
7	ENGINEERING	76	0.55	-
8	MATERIALS SCIENCE	69	0.41	6.6
9	GENERAL & INTERNAL MEDICINE	65	0.08	4.44
10	CARDIOVASCULAR SYSTEM & CARDIOLOGY	62	0.06	-

**Table 6:** The top 10 cited documents.

Rank	Frequency	Author	Journal	Article	Year	Burst
1	39	Sallis JF	CIRCULATION	Role of built environments in physical activity, obesity, and cardiovascular disease [30]	2012	8.37
2	29	Leal C	OBES REV	The influence of geographic life environments on cardiometabolic risk factors: a systematic review, a methodological assessment and a research agenda [31]	2011	4.94
3	29	Frank LD	AM J PREV MED	Obesity relationships with community design, physical activity, and time spent in cars [32]	2004	11.35
4	28	Saelens BE	MED SCI SPORT EXER	Built environment correlates of walking: a review [12]	2008	6.68
5	26	Papas MA	EPIDEMIOL REV	The built environment and obesity [33]	2007	6.75
6	26	Malambo P	PLOS ONE	Built environment, selected risk factors and major cardiovascular disease outcomes: a systematic review [34]	2016	9.95
7	24	Gordon-Larsen P	PEDIATRICS	Public parks and physical activity among adolescent girls [35]	2006	6.54
8	20	James Peter	Curr Epidemiol Rep	A review of the health benefits of greenness [36]	2015	4.95
9	20	Brook RD	CIRCULATION	Particulate matter air pollution and cardiovascular disease: an update to the scientific statement from the American Heart Association [37]	2010	4.66
10	20	Frank LD	AM J PREV MED	Linking objectively measured physical activity with objectively measured urban form: findings from SMARTRAQ [38]	2005	7.01

**Table 7:** Summary table of cluster information.

Cluster ID	Size	Silhouette	Mean (Year)	Label (LLR)
0	67	0.857	2007	walking; active commuting; epidemiology; risk assessment; exercise
1	63	0.844	2015	walkability; neighborhood; type 2 diabetes mellitus; heart disease; residence characteristics
2	58	0.837	2006	residence characteristics; obesity; body mass index; socioeconomic; mastery
3	55	0.752	2010	food environment; descriptive norms; cardiometabolic risk; air pollution; geographic information systems
4	54	0.949	2015	greenness; greenspace; green space; physical activity; noise
5	37	0.935	2010	endocrine system; indicators; cortisol; brain activity; forest
6	23	0.926	2009	youth; population; prevention; behavioral epidemiology; controlled studies
7	21	0.968	2013	particulate matter; air pollution; chronic disease prevention; electronic health records; exposure–response function
8	20	0.971	2000	environmental; adolescents; activity patterns; interventions; food prices
9	12	0.929	2007	expectations; aging; life expectancy; life table; accidents

Note: The silhouette value is the parameter used by CiteSpace software to evaluate the clustering effect. Specifically, the evaluation of clustering measures the homogeneity of the network. The closer the silhouette value is to 1, the higher the homogeneity of the network and the clustering results with high reliability are greater than 0.7.

## Data Availability

Not applicable.
